# Out of Control!? How Loss of Self-Control Influences Prosocial Behavior: The Role of Power and Moral Values

**DOI:** 10.1371/journal.pone.0126377

**Published:** 2015-05-29

**Authors:** Anne Joosten, Marius van Dijke, Alain Van Hiel, David De Cremer

**Affiliations:** 1 Department of Developmental, Personality, and Social Psychology, Ghent University, Ghent, Belgium; 2 Rotterdam School of Management, Erasmus University, Rotterdam, The Netherlands; 3 Judge Business School, University of Cambridge, Cambridge, United Kingdom; University of New South Wales, AUSTRALIA

## Abstract

Lack of self-control has been suggested to facilitate norm-transgressing behaviors because of the operation of automatic selfish impulses. Previous research, however, has shown that people having a high moral identity may not show such selfish impulses when their self-control resources are depleted. In the present research, we extended this effect to prosocial behavior. Moreover, we investigated the role of power in the interaction between moral identity and self-control depletion. More specifically, we expected that power facilitates the externalization of internal states, which implies that for people who feel powerful, rather than powerless, depletion decreases prosocial behavior especially for those low in moral identity. A laboratory experiment and a multisource field study supported our predictions. The present finding that the interaction between self-control depletion and moral identity is contingent upon people’s level of power suggests that power may enable people to refrain from helping behavior. Moreover, the findings suggest that if organizations want to improve prosocial behaviors, it may be effective to situationally induce moral values in their employees.

## Introduction

Research suggests that in order to display prosocial and cooperative behaviors, people require active self-control to override their automatic selfish impulses [1]. This suggestion may have important implications in the context of work organizations because prosocial employee behaviors like voluntary helping one’s supervisor and coworkers, speaking up to improve the way in which work is organized, and attempting to offer the best customer service possible all play a significant role in effective organizational functioning [2,3]. However, a variety of forces that are known to hamper and deplete self-control are omnipresent in work situations, such as the necessity to make many choices and decisions [4], overly long working hours that lead to sleep deprivation [5,6], and stress [7]. In other words, a number of factors that seem inherent to organizational life may constrain prosocial employee behavior, and therefore organizational effectiveness.

Yet, not everybody requires active self-control to display prosocial behavior. More specifically, people who have internalized moral values—as indexed by a high moral identity—may act in prosocial ways regardless of their level of self-control. This is an important theoretical idea because it presents a different perspective on the workings of automatic processes than most other studies, which usually assume that selfishness is automatically activated (e.g., [8–10]). However, internalized moral values have been argued to facilitate the self-regulation of moral behavior [11,12], which should explain why they can override automatic self-oriented processes.

Unfortunately, there is as yet little empirical evidence to substantiate these arguments in the context of prosocial behavior. The present research therefore focuses on the interaction between internalized moral values and self-control depletion in predicting voluntary prosocial behaviors. Research on negative and antisocial behaviors has shown that the combination of depletion and low moral identity increases antisocial behavior [13,14]. However, in the present paper we argue that selfishness by showing antisocial behavior is inherently different from selfishness by refraining from prosocial behavior. We argue that people need power to feel that they can refrain from helping others. People who feel powerful are more inclined to disregard others [15,16] and therefore more likely to deviate from prevailing norms [17]. We thus expect that power is likely to be a facilitator of the selfish state resulting from the combination of depletion and low moral identity.

In the following sections, we will first develop our argument regarding the relevance of self-control for the display of voluntary prosocial behaviors and the role of internalized moral values in this process. We develop our reasoning using the influential strength model of self-control (see [18] for an overview). Internalized moral values are analyzed in terms of theorizing on moral identity [19–21]. Then, we will develop our argument regarding the critical moderating role of power in this process. This will result in a hypothesis regarding a three-way interaction effect of self-control, moral identity and employee power on voluntary prosocial behavior.

### Theoretical background

#### Self-control, depletion, and prosocial behavior

Self-control refers to an individual’s ability to inhibit, override, or refrain from acting upon his/her impulses and desires [22–24]. The human capacity for self-control is extremely adaptive and enables people to follow society’s norms and rules [24,25]. In line with this, research has shown that self-control failures may lead to various behavioral problems that can be harmful to people and to social collectives, such as depression, aggression, the inability to manage finances, and theft. Conversely, successful self-control has been linked to numerous positive outcomes such as success at work, increased concentration, and an improved ability to cope with stress and problems (see [18] for an overview).

Research on self-control failures suggests that the capacity for self-control is a limited resource, which, with repeated use, can become depleted [26]. When self-control resources are depleted, performance on subsequent acts that require self-control may be impaired [18,26]. Self-control failures are thus more likely to emerge when an individual performs multiple acts that require self-control without rest or replenishment [26,27].

Importantly, the limited resource model of self-control may also have implications for our understanding of prosocial behavior. Specifically, it has been argued that displaying prosocial behavior and avoiding antisocial behavior requires self-control to override selfish impulses [1]. Indirect support for this idea is found in laboratory research that focuses on antisocial behavior showing that after an initial act that required self-control, people were more likely to cheat [9,13] and to act aggressively [28]. Research focusing on prosocial behavior, however, is scarce, if non-existent. We know of only one paper that addressed this issue but mostly in terms of prosocial intentions: DeWall and colleagues [1] showed that depletion reduced participants’ intention to help, but helping behavior was not included in the design. These findings suggest that people need self-control resources for prosocial behaviors to emerge. Interestingly, research suggests that having moral values (i.e., moral identity) facilitates the self-control of prosocial behavior [29]. That is, people with a high moral identity are more likely to have moral values readily accessible, even in situations that impair self-control. Below, we explicitly argue how moral identity may influence the self-regulation of prosocial behavior.

#### Moral identity

Moral identity reflects the degree to which people consider being a moral person an important part of their self-concept [19,20]. Moral identity has been conceptualized as a cognitive representation or schema of moral values, goals, traits, and behavioral scripts [20,29]. For people high in moral identity, this moral self-schema is more readily accessible and available for use than for people low in moral identity [20,30]. When activated, moral identity should have a strong influence on one’s cognition and behavior, as individuals have a strong tendency for self-consistency [19,31].

Consequently, moral identity is an important predictor of prosocial behavior [21] and has been associated with increased levels of self-reported volunteering [19], ethical leader behavior [32], an increased likelihood of making a donation [19,33], and charitable giving [34]. Additionally, moral identity has been linked to decreased levels of selfish behavior, such as less lying in business negotiations [20], lowered aggression on the football field [35], and less antisocial behavior among adolescents [36].

Important for the present purposes, moral identity may also facilitate the self-regulation of prosocial behavior in situations that constrain the availability of self-regulatory resources (e.g., self-control depletion). As argued above, people with a high moral identity have more readily accessible moral values than people with a low moral identity [29]. Consequently, people with a high moral identity should be especially likely to expend extra effort to self-regulate their prosocial behaviors. Over time, people with a high moral identity will thus more frequently implement prosocial behavior, resulting in more internalized and automatic enactment of prosocial behavior [12]. People with a high moral identity are thus likely to have their moral values more readily available, even in situations in which their self-control resources are depleted. We know of only two studies that offer some indirect support for this argument, but this support is offered in the realm of negative behavior. This research shows that depletion makes people low in moral identity more likely to show antisocial behavior, whereas this negative effect of depletion was absent among people high in moral identity [13,14]. In other words, the combination of depletion and a low level of moral identity represents a negative cocktail as evinced by the heightened levels of antisocial behavior.

However, findings obtained with negative behaviors cannot be straightforwardly extrapolated to (the non-display) of positive behavior. In philosophy, an important distinction is made between positive (i.e., do good for another) and negative duties (i.e., refraining from doing something morally bad; [37]). Importantly, Kant [38] argued that negative duties are more stringent than positive duties. In other words, refraining from negative behavior is considered more pressing than positive behavior, and therefore, negative behaviors are often regulated by state legislation [39]. Likewise, in organizations, refraining from antisocial and selfish behavior is regulated by formal sanction systems, whereas displaying prosocial behavior is often informal and more easy to implement because of its’ social desirability. Admittedly, the display of prosocial behavior might sometimes be restrained by, for example, formal organizational rules and regulations [40] or by the demands that are inherent in employees’ primary tasks [41]. However, helping others is often considered to be rewarding and these behaviors ‘feel good’ [42,43]. These behaviors are already stimulated at a young age [44]. Moreover, such behaviors are ‘the right thing to do’ and as such affirm one’s morality (see [45]). Thus, these behaviors are mostly regulated by informal norms rather than by explicit sanctioning systems.

Variations in the display of antisocial and prosocial behavior can thus *not* be expected to be symmetrical. As such, selfishness by showing antisocial behavior is inherently different from selfishness by refraining from prosocial behavior. One can thus not straightforwardly extrapolate the effects of factors that influence the display of negative and antisocial behaviors toward the non-display of positive and prosocial behaviors. Hence, it remains to be seen whether the interaction effect between moral identity and depletion on antisocial behavior generalizes to the display of prosocial behavior. As we argue below, it is likely that power is a facilitator of the selfish state resulting from the combination of low moral identity and depletion. In other words, it may be that people actually need power to feel that they can refrain from prosocial behavior.

#### Power as an inhibitor of prosocial behavior

Power is typically defined as one’s ability to administer and deny valuable resources or punishment to other people (e.g., [15,46,47]). Power is a central aspect of organizational contexts [48,49], and as such, can have a substantial impact on the emergence of selfish behaviors. Specifically, power has often been viewed as a corruptive force, influencing people to behave in self-interested ways [15,50–52]. A number of empirical studies have indeed suggested that people who experience power tend to focus on selfish impulses and subordinate the needs of others to their own desires (for overviews, see [15,16]). Moreover, the experience of power makes people less likely to empathize with someone else [53,54]. People who experience power are also less influenced by others and less likely to conform to prevailing norms [17]. In sum, it seems that people who feel powerful are inclined to disregard others in their behavior.

More recent research, however, suggests that the relation between power and self-interested behavior may be more complex [15]. Rather than directly influencing behavior, power may instead amplify the behavioral expression of individual predispositions [48,51,55,56]. Wisse & Rus [57], for example, found that people who experienced power displayed more antisocial behavior when they focused on their personal self than when they focused on their social self.

The finding that power magnifies inherent impulses is interesting in the context of moral identity and self-control depletion. Because the combination of a low moral identity and self-control depletion has been reported to increase antisocial behavior and, as such, can be considered to represent a cocktail of selfishness, power should be expected to be a magnifying factor. As we argued before, it is not possible to simply translate results found in the realm of negative behavior to positive behavior, and it therefore remains to be shown whether the combination of low moral identity and depletion leads to lower levels of prosocial behavior, or if power is a necessary facilitator of this effect. We expect the latter to be true for two reasons. First, prosocial behavior is usually displayed in high quality relationships such as workplace relationships. Power, however, may actually undermine this prevalence of prosocial behavior in high quality relationships. More specifically, power leads to an objectification of others, which transforms workplace relationships in exchange relationships, as such undermining prosocial behavior [56]. Second, while the display of positive behavior is enhanced by societal norms and education, high power undermines conformity [17], and therefore less helping behavior can be expected. In other words, people high in power may feel that they are in a position where they can get away with less helping behavior.

For people high in moral identity, on the other hand, depletion does not influence their level of selfishness as research suggests that high moral identifiers have their moral values more readily accessible even in situations of self-control depletion [13,14]. Because prosocial behavior is easy to implement and generally sustained by societal and organizational norms, we expect that people high in moral identity act in line with these societal norms irrespective of their level of depletion. In the same vein, one could also reason that power, as a facilitator of individual predispositions, may increase the prosocial behavior of people high in moral identity. Indeed, there is some research that indicates that people high in power who focus on moral or prosocial values show less antisocial behavior than those low in power [48,57]. Prosocial behavior is-unlike antisocial behavior- relatively easy to implement and sustained by societal and organizational norms. We expect that because of this high social acceptance of most prosocial behaviors, power will not lead to more prosocial behavior for high moral identifiers. That is, we expect that prosocial behavior is already part of the daily routine for people high in moral identity, and power is not likely to increase their helping behavior beyond this level.

#### Overview of predictions and studies

There is reason to believe that self-control depletion undermines the emergence of prosocial behaviors. However, internalized moral values in terms of a high moral identity facilitate the self-regulation of prosocial behavior, even in situations that impair self-regulation. In other words, depletion is likely to make people low in moral identity less prosocial, whereas depletion should have no effect on people high in moral identity. In the present research we expect that—contrary to the negative effects of depletion and low moral identity on antisocial behavior—power is a facilitator of the negative combination of depletion and low moral identity on prosocial behavior. It is likely that people may need power to feel that they can get away with refraining from prosocial behavior. Hence, we expected that power facilitates the interaction effect of depletion and moral identity on prosocial behavior. This leads to our Hypothesis, which implies a three-way interaction between depletion, moral identity and power. In particular, when power levels are high, a combination of depletion and low moral identity lead people to refrain from prosocial behavior, whereas no such an effect is expected when power levels are low. The present study’s Hypothesis therefore states that:

*The negative effect of depletion on prosocial behavior among people low in moral identity is restricted to people high*, *rather than low in power*.

We tested this Hypothesis in two studies. Study 1 was a controlled laboratory experiment in which participants’ power and level of depletion were manipulated. We measured the participant’s level of moral identity independent from the experimental situation. The dependent variable in this study was the extent to which the participants helped another person who was in need.

The controlled setting in Study 1 makes it possible to draw causal conclusions, but it does not tell us much about the relevance of the processes that we set out to study in actual organizational contexts. Therefore, Study 2 was conducted in an organizational setting, using a multisource design. We asked employees of various organizations to indicate their level of depletion, their moral identity, and their power in the organization using well-established measures. To avoid potential common method and self-presentation biases [58] we asked a colleague to indicate the focal employee’s level of prosocial behavior. We operationalized prosocial behavior as organizational citizenship behavior (OCB). OCB is an important and commonly used index of prosocial employee behavior because it describes various types of discretionary, extra-role behaviors that contribute to effective organizational functioning but that are not explicitly required [59].

## Study 1

### Method

#### Ethics statement

Ethics approval for Study 1 was formally waived by the ethical committee of the Faculty of Psychological and Educational Sciences (FPPW), Ghent University, as this research was performed in adherence with the ethical protocol of the university. All participants gave their formal, written consent, and were fully debriefed after the experiment. Participants participated voluntary and they could quit the experiment at any time without negative consequences. All data was analyzed and stored anonymously.

#### Participants and design

Eighty-four undergraduate students from a medium-sized Belgian university participated in this study (Note: Three respondents were not included in the analyses because they did not follow the instructions of the power manipulation. Inclusion of these three respondents in our analyses did not change any of the results. Most importantly, the predicted three-way interaction remained significant, β = .29, *p* = .01). The average age of participants was 18.95 years (*SD* = 2.11), and 89.3 percent were women. The participants were recruited through an online sign-up system and received partial course credit for their participation. Participants were randomly assigned to one condition of a 2 (depletion versus no depletion) x 2 (high versus low power) between subjects design. Participants’ moral identity was assessed prior to the experimental manipulations, creating an additional continuous between subjects variable.

#### Moral identity measure

Participants responded to an online questionnaire including demographic information and a measure of moral identity 24 hours before the actual experiment. We used Aquino and Reed´s [19] instrument to measure participants’ moral identity. Following Aquino and Reed [19], and in line with our theoretical ideas, we relied on the Internalization dimension of this instrument (i.e., the extent to which people find morality an important aspect of who they are) and disregarded the Symbolization subscale (which measures the extent to which people want to appear as a moral person). The Internalization subscale has been proven to be the most stable and robust predictor of moral behavior [29,34]. In line with Aquino and Reed’s [19] procedure, the following instructions were given: “Listed below are some characteristics that might describe a person: Caring, Compassionate, Fair, Friendly, Generous, Helpful, Hardworking, Honest, and Kind. The person with these characteristics could be you or it could be someone else. For a moment, visualize in your mind the kind of person who has these characteristics. Imagine how that person would think, feel, and act. When you have a clear image of what this person would be like, answer the following questions.” Participants then responded to the five Internalization items on a 7-point scale. Sample items from this scale are: “It would make me feel good to be a person who has these characteristics” and “Having these characteristics is an important part of my sense of self” (1 = *totally disagree*; 7 = *totally agree*; Cronbach’s α = .72; *M* = 6.18, *SD* = 0.60).

#### Experimental procedure

Upon arrival at the laboratory, participants were seated in separate cubicles, each equipped with a personal computer. All communication took place via this computer.

First, participants were introduced to the power manipulation, taken from Galinsky and colleagues [51] that served to prime high versus low power. Participants were asked to recall a particular situation in their lives. Participants in the high power condition wrote about “a particular situation in which you had power over another individual or individuals”. Participants in the low power condition wrote about “a particular situation in which someone else had power over you.”

Following the power manipulation, participants responded to the manipulation checks using two items (adapted from [60]): “How powerful did you feel in the situation you recalled” and “How much power did someone else have over you in the situation you recalled” (reversed; 1 = *not at all*; 7 = *very much so*).

Participants then completed the depletion task (taken from Baumeister et al., 1998). This task has proven to be successful as a manipulation of self-control depletion in a number of studies (e.g., [26,61,62]). In the first part, participants were instructed to indicate each instance of the letter *e* in a text (i.e., by clicking each *e* with the computer mouse). Participants received visual feedback whenever they clicked an *e* (i.e., a highlighted circle around the corresponding *e*), and were given five minutes to complete the task. This first phase is relatively easy and is used to establish a strong habitual response for scanning and indicating every *e*. In the second part of the task, participants either continued indicating *e’*s using the same rule as before (*no depletion* condition), or they were given the instruction to indicate each *e*, except for the ones followed by a vowel, or those with a vowel preceding the *e* by two letters (*high depletion* condition). For participants in the high depletion condition, overriding the response of scanning for and indicating every *e* is known to require more regulatory resources than for participants in the low depletion condition (who did not need to override a habitual response).

The effectiveness of the self-control depletion manipulation was assessed using two items: “The second task was hard” (taken from [63]), and “The second task was habit-breaking” (1 = *not at all*; 7 = *very much so*; taken from [1]).

#### Helping measure

After the experimental tasks, participants were told that there were several PhD students in need of participants for their experiments that lasted usually somewhere between 5 and 60 minutes. Participants were asked whether they would be willing to participate. We emphasized to the participants that it was not possible to reward them for their participation in these additional studies, and that they would be contacted by an experimenter to set a date and time that would suit them best. Then, participants indicated how much time they would help (i.e., number of donated minutes) or by indicating that they would not help (coded as 0 donated minutes; see e.g., [64,65] for similar ways to measure prosocial behavior). Subsequently, participants were fully debriefed.

### Results

#### Manipulation checks

A 2 (depletion versus no depletion) x 2 (high power versus low power) Analysis of Variance (ANOVA) showed that participants in the high power condition considered themselves more powerful in the recalled situation than participants in the low power condition (*M* = 4.81, *SD* = 1.40 vs. *M* = 2.14, *SD* = 1.00, respectively), *F*(1, 80) = 99.24, *p* < .001, η^2^ = .55. These participants also disagreed more with the statement that someone else had power over them than participants in the low power condition (*M* = 4.55, *SD* = 1.23 vs. *M* = 5.29, *SD* = 1.15, respectively), *F*(1, 80) = 8.17, *p* = .01, η^2^ = .09. No other main or interaction effects were significant.

Additionally, two independent judges rated how powerful the participants were in the recalled situations on a 7-point scale (1 = *not at all powerful*; 7 = *very powerful*). The inter-rater reliability was high (Intraclass correlation coefficient [ICC] = .90) and ratings were averaged to assess the effectiveness of the power manipulation. A 2 (depletion versus no depletion) x 2 (high versus low power) ANOVA showed that participants in the high power condition were rated more powerful in the described situation than participants in the low power condition (*M* = 4.85, *SD* = 0.58 vs. *M* = 3.20, *SD* = 0.90, respectively), *F*(1, 80) = 99.34, *p* < .001, η^2^ = .55. No other main or interaction effects were significant.

A 2 (depletion versus no depletion) x 2 (high versus low power) ANOVA indicated that depleted participants rated the depletion task as harder than non-depleted participants (*M* = 4.88, *SD* = 1.33 vs. *M* = 3.60, *SD* = 1.50, respectively), *F*(1, 80) = 17.62, *p* < .001, η^2^ = .18. These participants also found the task more habit-breaking than non-depleted participants (*M* = 5.05, *SD* = 1.38 vs. *M* = 3.95, *SD* = 1.46, respectively), *F*(1, 80) = 12.40, *p* = .001, η^2^ = .13. No other main or interaction effects were significant.

We also conducted regression analyses in which the manipulation checks were predicted by the depletion manipulation, power manipulation, participants’ moral identity, and the corresponding interaction terms. These analyses produced similar results to those presented in the main text. Specifically, power increased how powerful participants felt, β = .75, *p* < .001, and decreased reported feelings of powerlessness, β = -.30, *p* = .01. Furthermore, participants in the high power condition were rated significantly more powerful than participants in the low power condition, β = .83, *p* < .001. Finally, depletion increased ratings of how hard, β = .43, *p* < .001, and habit-breaking the task was, β = .35, *p* = .001. In none of the analyses, other main or interaction effects were significant.

### Helping behavior

Our measure of helping behavior (i.e., asking participants to donate their time for participation in additional studies) was positively skewed (*M* = 21.31, *SD* = 16.79). This resulted because a significant number of cases (*N* = 16) clustered at the lower limit (i.e., helping out for 0 minutes, to indicate that they did not want to display prosocial behavior). Skewed distributions can result in the violation of OLS assumptions. We therefore conducted a Tobit regression (see [66]), which was specifically developed for variables with a lower (or upper) limit and a concentration of observations at this limiting value.

To test our hypothesis, we thus conducted a Tobit regression analysis in which helping behavior was predicted by the depletion manipulation, moral identity, the power manipulation, all the two-way interactions among these three variables, and finally, the three-way interaction. Following Aiken and West [67], the interaction terms were based on the mean-centered scores of moral identity and effect coded scores of depletion and power.

**Fig 1:**
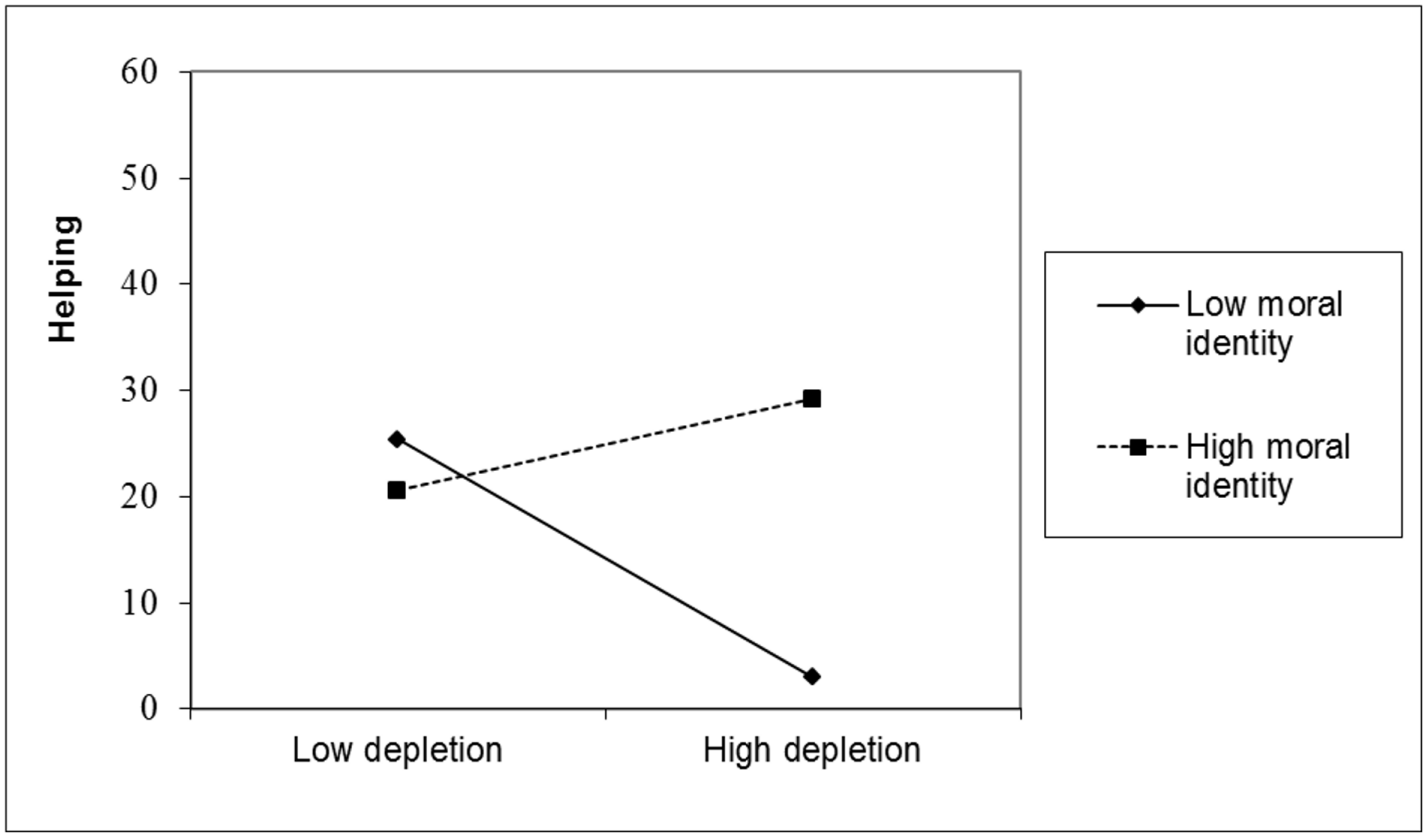
Helping as a Function of Depletion and Moral Identity for Participants High in Power.

Table 1 shows the results of the Tobit regression analysis. Of most importance, the predicted three-way interaction was significant, β = .34, *p* = .004. To analyze this interaction in more detail, we used simple slope analyses [67]. Fig 1 shows that, consistent with our predictions, among participants who were primed with high power, depletion significantly decreased helping behavior for those low in moral identity (one *SD* below the mean), β = -.55, *p* = .02, but not for those high in moral identity (one *SD* above the mean), β = .21, *p* = .33.

**Table 1:** Results of Hierarchical Regression Analysis for Helping in Study 1.

Variables	*B*	*SE B*	β
Self-control depletion (SD)	-1.26	2.11	-.06
Moral identity (MI)	5.24	3.90	.15
Power (P)	-0.34	2.11	-.02
SD x MI	1.24	3.90	.04
SD x P	-2.14	2.11	-.11
MI x P	3.66	3.90	.11
SD x MI x P	11.55	3.92	.34**

*Note*. Final model: -2 log likelihood = -311.39, χ^2^ (7) = 11.29, *p* = .13. *B* = unstandardized regression coefficient; β = standardized regression coefficient. For the self-control depletion manipulation, -1 denotes no self-control depletion; 1 denotes self-control depletion. For the power manipulation, -1 denotes low power; 1 denotes high power.

** *p* < .01.

**Fig 2:**
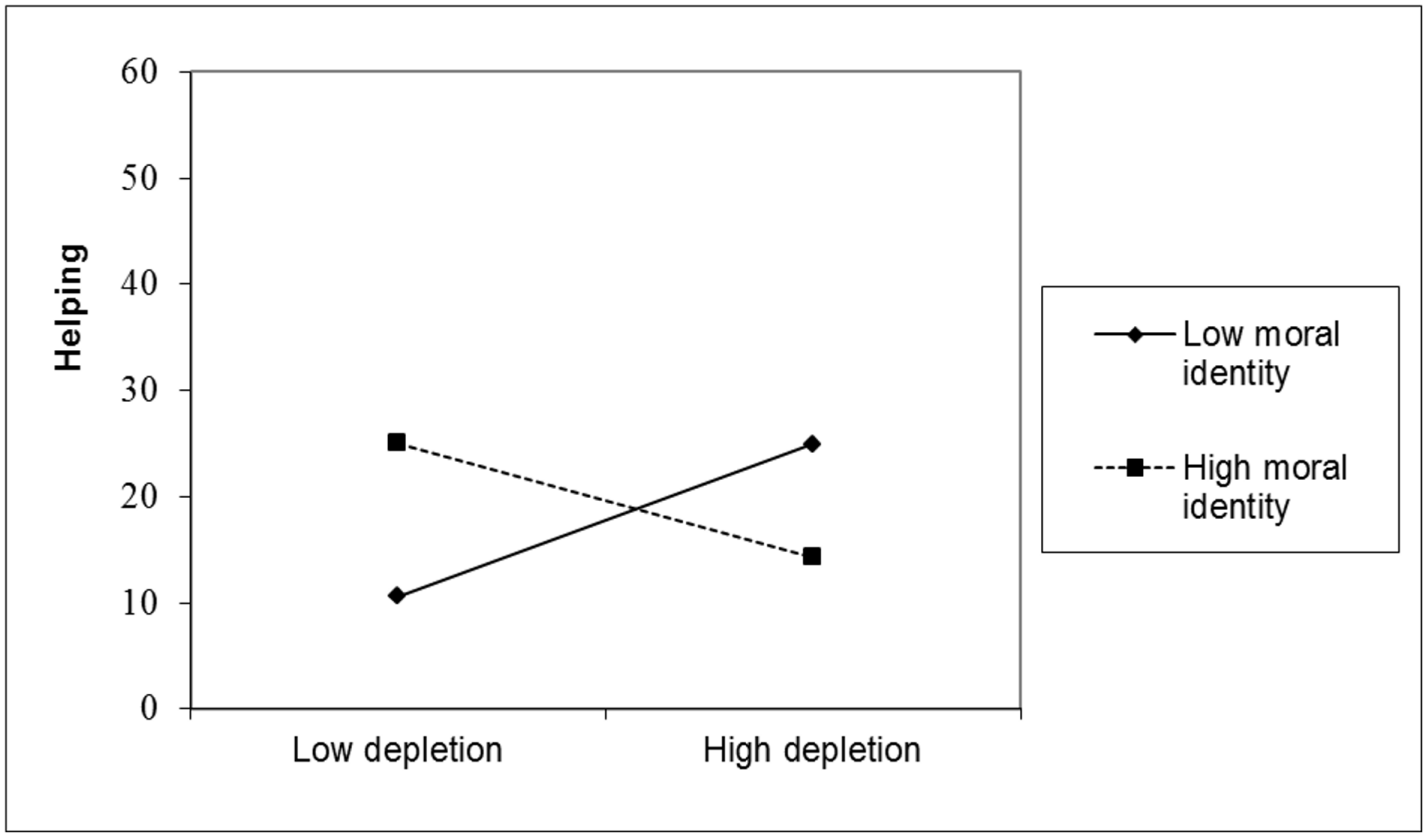
Helping as a Function of Depletion and Moral Identity for Participants Low in Power.

Yet, for participants who received the low power prime (see Fig 2), depletion did not significantly influence helping behavior for those low in moral identity (one *SD* below the mean), β = .35, *p* = .09, or for those high in moral identity (one *SD* above the mean), β = -.26, *p* = .24.

We also conducted OLS regression analyses. These analyses produced similar results as the Tobit regression analyses. Most importantly, the predicted three-way interaction was significant, β = .28, *p* = .02.

### Summary and conclusion

The results of Study 1 show that, in line with theoretical predictions [12] and our Hypothesis, among participants who felt high in power, depletion reduced prosocial behaviors for those low (as opposed to high) in moral identity, whereas this interaction effect between depletion and moral identity did not occur for those who felt low in power.

## Study 2

Study 1 provided causal evidence for our proposed ideas, but the setup limited us to the use of students as participants in a laboratory setting. Study 2 was designed to test our predictions in an organizational setting. Rather than priming power and manipulating depletion, we measured employees’ sense of power in the organization and their level of depletion in addition to their moral identity. To avoid potential common method and self-presentation biases we asked colleagues of the respondents to rate the respondent’s prosocial behavior [58].

### Method

#### Ethics statement

Ethics approval for Study 2 was formally waived by the ethical committee of the FPPW, Ghent University, as this research was performed in adherence with the ethical protocol of the university. We used a research agency to recruit our respondents, who gave their consent upon enrolling this research panel to use their data for research purposes. Moreover, a “double active opt-in” method was used, meaning that all respondents gave their consent by actively and voluntarily agreeing to take part in our research. Before starting the questionnaire, all respondents were provided with information on the purpose and the content of the research. Respondents were informed that all data would be analyzed and stored anonymously and that they could quit the questionnaire at any moment.

### Sample and procedure

We recruited respondents via a Dutch research panel. We asked potential respondents to respond to our survey and also to invite a coworker to respond to some items. A total of 893 panel members agreed to fill out the questionnaire as focal employee and 94 of these focal employees also found a colleague willing to fill out the questionnaire. The focal employees (i.e., panel members) received credit points that would allow them to receive certain gifts (e.g., tickets for the movies). Colleagues participated in a lottery in which they could win an Ipad or one of five €20 gift certificates. Because we relied on colleague ratings of the focal employee’s behavior, the number of respondents included in our analyses consisted of 94 employees and 94 matched colleagues.

Focal employees who could be included in the analyses (i.e., because they had a coworker who was also willing to participate) did not differ from focal employees who could not be included in the analyses with regard to their mean level on the demographic variables and focal predictors. There was one exception: focal employees who could be included worked longer in their current organization than focal employees who were not included. This is most likely because longer tenure increases the likelihood of developing social connections with colleagues. This should make it easier to find a coworker willing to participate.

In addition, we also tested whether the correlations between the study variables were significantly different between included and not included employees. The correlations between the study’s variables (Bonferroni corrected) did not differ between the two groups of focal employees. These analyses give us little reason to think that selection biases impacted our results and conclusions.

Of the focal employees, 55 were male and 39 were female. The mean age was 44.13 years (*SD* = 11.37). One percent had only lower education (primary school), 17% had high school only, 26% had followed up on this with vocational education, 36% had a bachelor’s degree, and 20% had a master’s degree. The focal employees worked on average for 12.83 years (*SD* = 10.80) in their current organization.

The matched group of colleagues included 47 males and 47 females. The mean age was 42.96 years (*SD* = 10.98). One percent had only lower education (primary school), 19% had high school only, 30% had followed up on this with vocational education, 43% had a bachelor’s degree, and 7% had a master’s degree. The colleagues worked on average for 10.72 years (*SD* = 9.27) in their current organization.

### Measures

We measured moral identity using the same internalization subscale of the moral identity measure [19] as in Study 1 (1 = *not at all*; 5 = *very much so*; Cronbach’s α = .77; *M* = 4.02, *SD* = 0.70).

To assess focal employees’ levels of depletion, we used the 2-item measure from Muraven and colleagues [27]. Focal employees indicated how much they agreed or disagreed with: “I often feel as if I have low energy,” and “I often feel as if things are taking a lot of effort” (1 = *strongly disagree*; 5 = *strongly agree*; Cronbach’s α = .72; *M* = 2.29, *SD* = 0.93).

Power of the focal employee was measured using the 8-item instrument developed by Anderson and Galinsky ([50]; see [68] for extensive validation evidence). Focal employees responded to items such as “Even if I voice them, my views have little sway in the organization” (reverse scored), and “If I want to, I get to make the decisions in the organization” (1 = *strongly disagree*; 5 = *strongly agree*; Cronbach’s α = .77; *M* = 3.51, *SD* = 0.89).

We operationalized prosocial behavior of the focal employee using the 19-item OCB measure of Moorman and Blakely [69]. To assess OCB, *colleagues* of the focal employees were asked to rate the focal employees on actions such as “voluntarily helps new employees settle into the job,” “often motivates others to express their ideas and opinions”, “performs his/her job duties with extra-special care,” and “actively promotes the organization’s products and services to potential users” (1 = *strongly disagree*; 5 = *strongly agree*; Cronbach’s α = .91; *M* = 3.87, *SD* = 0.52).

### Results

#### Descriptive statistics and intercorrelations

Table 2 presents the means, standard deviations, and correlations between the Study 2 variables.

**Table 2:** Descriptive Statistics and Intercorrelations of Study 2 Measures.

Variable	*M*	*SD*	1	2	3	4	5	6	7	8	9	10
1. Self-control depletion	2.29	0.93	(.72)									
2. Moral identity	4.02	0.70	-.19	(.77)								
3. Power	3.51	0.77	-.23*	.30**	(.89)							
4. OCB (colleague rating)	3.87	0.52	-.19	.36**	.27**	(.91)						
5. Age (focal)	44.13	11.37	-.22*	-.15	-.03	-.04						
6. Gender (focal)	1.41	0.50	-.05	.13	.15	.20	.04					
7. Tenure (focal)	12.83	10.80	-.11	-.14	-.09	.01	.66**	-.03				
8. Education level (focal)	3.57	1.03	-.05	.25*	.18	.09	-.12	-.09	-.13			
9. Age (colleague)	42.96	10.98	-.12	-.17	.00	-.18	.32**	.06	.11	-.19		
10. Gender (colleague)	1.50	0.50	-.20	.29**	.14	.27**	-.12	.67**	-.13	.10	-.19	
11. Education level (colleague)	3.36	0.91	-.08	.27**	.31**	.07	-.04	-.15	.03	.64**	-.22*	.05

*Note*. N = 94. Internal reliabilities (coefficient alphas) are provided in parentheses on the diagonal. For gender, 1 denotes males, 2 denotes females.

* *p* ≤ .05.

** *p* ≤ .01.

### Hypothesis test

To test our hypothesis, we conducted a hierarchical regression analysis with colleague ratings of OCB serving as the dependent variable. Age, gender, tenure, and education level of the focal employees, and, age, gender, and education level of the colleagues were entered as control variables in the first step of the regression. Depletion, moral identity, and power were entered in the second step of the regression. The two-way interactions between depletion, moral identity, and power were entered in the third step of the regression. The three-way interaction was entered in the fourth step. Interaction terms were based on mean-centered scores of the independent variables [67].

**Fig 3:**
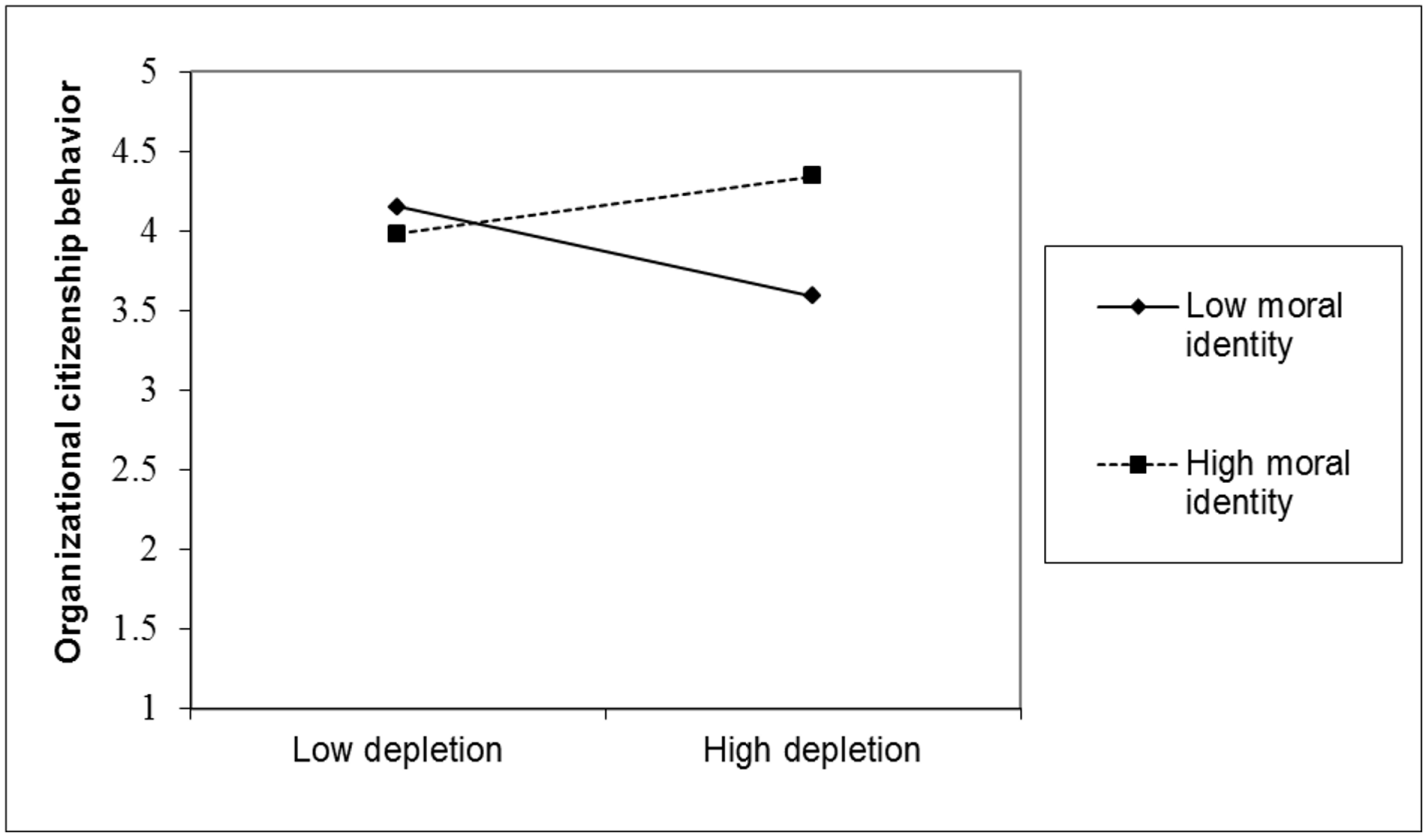
OCB (Coworker Rating) as a Function of Depletion and Moral Identity for High Power Employees.

Table 3 shows the results of the hierarchical regression analysis. Of most importance and in line with our Hypothesis, the predicted three-way interaction was significant, β = .24, *p* = .02. We used simple slope analyses [67] to analyze this interaction further. Fig 3 shows that, among high power employees, depletion significantly decreased OCB for those low in moral identity (one *SD* below the mean), β = -.95, *p* < .001. However, for those high in moral identity (one *SD* above the mean) depletion did not decrease OCB, β = .17, *p* = .35.

**Table 3:** Results of Hierarchical Regression Analysis for OCB in Study 2.

Variables	Step 1	Step 2	Step 3	Step 4
Age of focal employee	-.03	-.05	-.09	-.08
Gender of focal employee	.10	.09	.07	.07
Tenure of focal employee	.08	.12	.16	.15
Education level of focal employee	.06	.06	.07	.06
Age of colleague	-.14	-.16	-.12	-.10
Gender of colleague	.17	.07	.08	.11
Education level of colleague	.00	-.13	-.06	-.07
Self-control depletion (SD)		-.10	-.04	-.09
Moral identity (MI)		.26	.22*	.23*
Power		.19	.16	.15
SD x MI			.33**	.41***
SD x Power			-.29**	-.31**
MI x Power			-.03	.05
SD x MI x Power				.25*
*R* ^*2*^	.10	.23	.34	.38
*R* ^*2*^_*adj*_	.03	.13	.23	.27
*R* ^*2*^_*change*_	.10	.13**	.11**	.04*
*F*	1.35	2.42*	3.14**	3.45***

*Note*. Table presents Beta coefficients. For gender, -1 denotes males, 1 denotes females.

* *p* < .05.

** *p* < .01.

*** *p* < .001.

**Fig 4:**
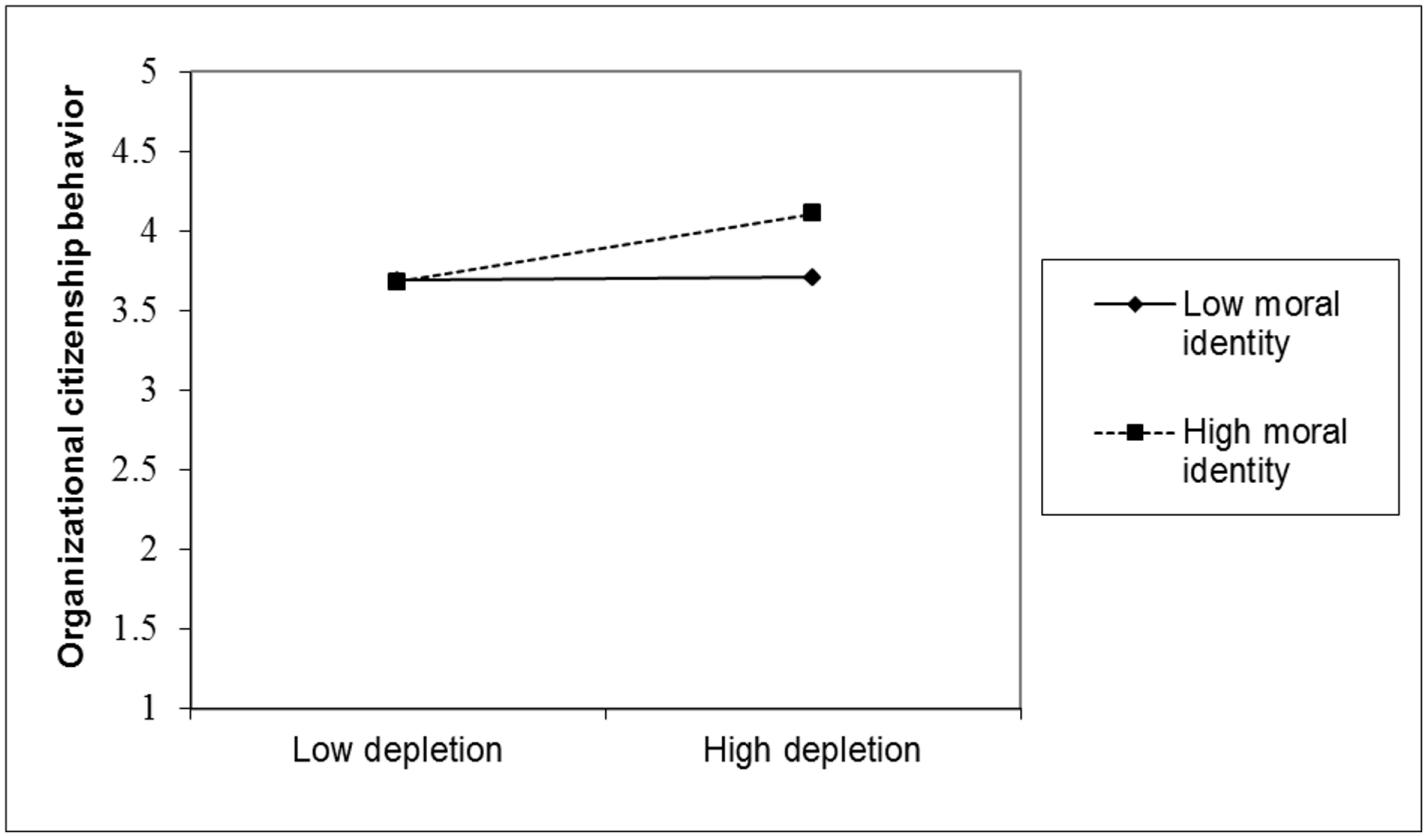
OCB (Coworker Rating) as a Function of Depletion and Moral Identity for Low Power Employees.

Fig 4 shows that, for low power employees, depletion had no effect on OCB for those low in moral identity (one *SD* below the mean), β = .02, *p* = .89. Unexpectedly, depletion increased OCB for those high in moral identity (one *SD* above the mean), β = .41, *p* = .050. However, given the fact that the interaction between moral identity and self-control depletion was not significant among employees low in power, and given that we did not obtain this result in Study 1, the results of this analysis should be interpreted with caution.

### Supplemental analyses

We followed Spector and Brannick’s [70] suggestion and repeated our analyses without the control variables as predictors in the equations. This analysis led to similar conclusions to those presented previously. Most importantly, the predicted three-way interaction was significant, β = .24, *p* = .02.

### Summary and conclusion

The results of Study 2 supported our prediction. We found the hypothesized interaction between moral identity and depletion for employees high in power, but not for employees low in power. More specifically, depletion reduces prosocial behaviors among employees low in moral identity if those employees feel high in power, but not if they feel low in power. The prosocial behavior of employees high in moral identity, on the other hand, was not influenced by depletion, whether they felt high in power or not. It thus seems that employees with a high moral identity have their moral values more readily accessible, even when their self-control resources are depleted and irrespective of their power level.

## General Discussion

A laboratory experiment and a multisource field study consistently showed an interaction between depletion and moral identity for people high in power, but not for people low in power. In the following sections we discuss the implications and limitations of these findings.

### Theoretical implications

The obtained three way interaction between self-control depletion, moral identity and power has theoretical implications for each of the constituting factors of this third order effect. It enhances, first of all, our understanding of the role of self-regulation in the display of prosocial behavior. In fact, most previous studies focused on effects of depletion on subsequent task persistence or negative and antisocial behavior [13,28,71]. To date, indirect evidence for the effect of depletion on prosocial behavior is offered only by DeWall and colleagues [1] who showed that depletion decreases prosocial *intentions*. Hence, our research is (at least to our knowledge) the first to show that regulatory depletion has an effect on prosocial *behavior*. These findings are important because our results indicate that especially people who feel powerful and are low in moral identity are likely to show less prosocial behavior as a result of regulatory depletion. At the same time, however, people high in power are likely to serve as a source of ethical guidance by means of social learning [72,73]. That is, if someone in power does not act in ethical ways, employees are likely to follow his or her lead [74].

Most importantly, the present findings offer corroborative evidence for the idea that the effect of situations that constrain cognitive capacity (e.g., self-control depletion) on prosocial behavior depends not only on one’s level of moral identity, but also on one’s sense of power. That is, self-control depletion leads to a decrease in prosocial behavior among people low in moral identity, but only when they feel powerful. Our reasoning for this is that prosocial behavior is fairly easy to implement because of its social desirability and it thus seems that people need power to feel that they can refrain from prosocial behavior. Research in the realm of antisocial behavior, however, has shown that the effect of self-control depletion on antisocial behavior depends solely on one’s level of moral identity [13,14]. That is, depletion increases antisocial behavior among people low in moral identity, irrespective of their power level. The self-regulation of prosocial behavior, on the other hand, is dependent upon people’s level of power. In other words, depletion reduces prosocial behavior among people low in moral identity, only if they experience power. Taking all these results together, it is clear that the display of prosocial intentions relies on processes that are qualitatively different from suppressing antisocial and selfish impulses (e.g., [75]).

The results of the present study also have implications for our understanding of what power tells us about the differences between not helping someone and hurting someone. In the introduction we argued that refraining from antisocial behavior is considered as more pressing than prosocial behavior [38]. That is, antisocial behavior is usually regulated by formal sanctioning systems, which are known to make people focus on the exchange characteristics of a situation [76,77]. Similarly, power is also likely to make people focus on the exchange characteristics of a situation, because people who experience power tend to objectify others [56]. It thus seems that similar processes that underlie the emergence of antisocial behavior, also play a role in the behavior of people high in power. Prosocial behavior, on the other hand, is regulated more informally because of its social desirability. Prosocial behavior is generally sustained by social and organizational norms, and adherence to these norms is fairly easy. The present study thus indicates that power is needed to obtain the same results for prosocial behavior as for antisocial behavior (i.e., the negative effect of self-control depletion for people low in moral identity; see [13,14]).

Our findings are also informative for the study of moral identity. Among people high in moral identity, self-control depletion and power do not necessarily hamper the self-regulation of prosocial behavior. This finding suggests that, in line with Gino and colleagues [13] and Joosten and colleagues [14], people high in moral identity have their moral values accessible irrespective of their level of depletion.

Our research has also some implications that are relevant for the power literature. Past research has, on the one hand, often shown that power can make people more selfish (for overviews see [15,16]). However, on the other hand, some studies suggest that this undermining effect on selfishness does not necessary result from having high power in itself [15,51]. As a solution to these diverging findings, it has been proposed that power in itself does not make people selfish but that it acts as a catalyst in facilitating the behavioral expression of internal states [60,78]. This indicates that power is not inherently corruptive, but rather a facilitator of the behavioral expression of internal states (in our case: the toxic cocktail of depletion and low moral identity). The present research adds to this literature, showing that the facilitating effect of power on internal states (i.e., low moral identity) is contingent upon third variables as well (i.e., self-control depletion).

### Practical implications

The present research also offers some practical implications for organizations. It seems to be the case that particularly employees who feel powerful are vulnerable to the effects of self-control depletion on prosocial behaviors. At the same time, it is especially important for employees high in power to behave in prosocial ways as they form an important source of vicarious learning [73]. For these employees, the negative effects of self-control depletion on prosocial behavior seem to apply particularly among those low in moral identity. Fortunately, research indicates that it is possible to situationally increase the accessibility of moral identity [29,79]. Combined with the present results, this entails a promising implication for organizations. Situational interventions aimed at stimulating moral identity are thus likely to make employees who feel high in power behave in prosocial ways. Such interventions can consist of the stimulation of a clear ethical climate. Moreover, social learning is enforced by ensuring that employees high in power act in moral ways, by which interventions aimed at increasing morality have positive implications for people low in power [74,80,81].

Another practical implication of the present findings is that on the one hand, high power makes employees particularly vulnerable to the effects of self-control depletion on prosocial behaviors, while, on the other hand, power also comes with heavy workloads, and numerous choices and decisions each day. Importantly, high stress levels [7], overly long working hours that may lead to sleep deprivation [5,6], and the necessity to make many choices and decisions [4], all constitute factors that are known to lead to self-control depletion. Organizations should thus be aware that overloading their employees in this respect could also reduce the prevalence of prosocial behaviors, at least among employees with a low moral identity and a high sense of power. Similarly, employees who feel high in power should also be aware that their cognitive state could affect their own behavior.

One could assume from our results that employees who feel low in power are not vulnerable to the effects of self-control depletion on selfish behaviors. It is, however, important that organizations and employees realize that this only holds for the emergence of prosocial behaviors. That is, our findings indicate that for employees low in power, depletion does not reduce prosocial behaviors for those low in moral identity. There are, however, studies in the realm of negative behavior that show that self-control depletion makes people low in moral identity more likely to show antisocial behavior [13,14]. Even though these studies did not compare high and low power, the results from these studies should nevertheless be taken into consideration.

### Strengths, limitations and suggestions for future research

A major strength of this article lies in the use of diverse methods to test our hypothesis. The laboratory experiment (Study 1) permits us to draw causal inferences with regard to the interactive effects of power, self-control depletion and moral identity on prosocial behavior. The subsequent multisource field study (Study 2) allowed us to investigate whether the hypothesized effects are also relevant in organizational settings. Furthermore, the multisource setting made it possible to control for common method and self-presentation biases [58].

A potential limitation is that the sample sizes in both Study 1 and Study 2 are relatively small and that this could potentially harm the validity of our results. We did, however, replicate the findings in an experimental setting (Study 1) and in a multisource field setting (Study 2), which reinforces the reliability and validity of our results. However, even though we believe that our results are valid and reliable, replications are necessary to further prove the validity of our findings.

In Study 2, we relied on colleague ratings of OCB. Our reliance on a single source to measure OCB may pose a threat to the validity of our findings, because of the discretionary nature of OCB [82]. That is, OCB consists of many different behaviors, and it is not unlikely that the colleagues witnessed only part of these behaviors. It may thus be that our reliance on a single source measure does not fully capture the unique variance present in citizenship behaviors. Future research could address this possible shortcoming by measuring OCB via various sources (e.g., comparing self and other ratings, or by combining various other ratings).

Another strength of the present article is that self-control depletion was manipulated in Study 1, whereas it was measured in Study 2. Although it can be argued that the manipulation of self-control depletion represents a more dynamic representation of self-control depletion than the more trait oriented measure, similar results were obtained. This apparent consistency strengthens our beliefs that it is possible to capture self-control depletion with a trait oriented measure in the field. These results also corroborate previous research that combined self-control depletion manipulations and measures, which shows clear consistency between these two operationalizations of self-control depletion [4,14]

Readers could wonder whether there are situations in which power may increase the prosocial behavior of people high in moral identity. In our research we focused on informal, effortless helping behavior. As noted in the introduction, prosocial behavior might sometimes be restrained by organizational rules and regulations or by demands inherent in employees’ primary tasks [40,41]. In these cases, prosocial behavior is thus likely to be more effortful and less socially desirable, and may have as a result that high moral identifiers need power to act in line with their moral values.

## Concluding remarks

Research focusing on the social effects of depletion presents us with a rather cynical view of human nature. Lack of self-control results in selfishness [8–10], and is also likely to undermine the emergence of prosocial behaviors. Yet, other studies show that depletion makes only people low in moral identity more selfish, while no such an effect of depletion was obtained among high moral identifiers. We argued that one cannot simply extrapolate the effects of factors that influence the display of antisocial behavior to the non-display of prosocial behavior, and that one may need power to refrain from prosocial behavior. In line with this, we showed that the moderating role of moral identity on the effects of depletion is present among people high in power, and not among people low in power.
